# Desktop Virtual Reality Versus Face-to-Face Simulation for Team-Training on Stress Levels and Performance in Clinical Deterioration: a Randomised Controlled Trial

**DOI:** 10.1007/s11606-022-07557-7

**Published:** 2022-05-02

**Authors:** Sok Ying Liaw, Sutini, Wei Ling Chua, Jian Zhi Tan, Tracy Levett-Jones, Balakrishnan Ashokka, Terry Ling Te Pan, Siew Tiang Lau, Jeanette Ignacio

**Affiliations:** 1 Alice Lee Centre for Nursing Studies, Yong Loo Lin School of Medicine, National University of Singapore, Level 2, Clinical Research Centre, Block MD11 10 Medical Drive, Singapore, 117597 Singapore; 2 Woodlands Health Campus, Singapore, Singapore; 3 School of Nursing & Midwifery, Faculty of Health, University of Technology Sydney, Ultimo, Australia; 4 Department of Anaesthesia, Yong Loo Lin School Medicine, National University of Singapore, Singapore, Singapore

**Keywords:** clinical deterioration, interprofessional education, simulation, stress, virtual reality

## Abstract

**Background:**

Simulation-based education can equip healthcare providers with the ability to respond to and manage stressors associated with rapidly deteriorating patient situations. However, little is known about the benefits of using virtual reality (VR) for this purpose.

**Objective:**

To compare between desktop VR and face-to-face simulation in stress responses and performance outcomes of a team-based simulation training in managing clinical deterioration.

**Design:**

A randomised controlled study

**Method:**

The study was conducted on 120 medical and nursing students working in interprofessional teams. The teams were randomly assigned to participate in a 2-h simulation using either the desktop VR or face-to-face simulation with simulated patient (SP). Biophysiological stress response, psychological stress, and confidence levels were measured before and after the simulation. Performance outcomes were evaluated after the simulation using a deteriorating patient scenario.

**Results:**

The systolic blood pressure and psychological stress response were significantly increased among participants in VR and SP groups; however, no significant differences were found between the groups. There was also no significant difference in confidence and performance outcomes between participants in the VR and SP groups for both medical and  nursing students. Although the psychological stress response was negatively correlated (*r* = −0.43; *p* < 0.01) with confidence levels, there was no association between stress response and performance score.

**Conclusion:**

Despite being less immersive, the desktop VR was capable of inducing psychological and physiological stress responses by placing emotional, social, and cognitive demands on learners. Additionally, by ensuring close alignment between the simulation tasks and the clinical tasks (i.e. functional fidelity), the desktop VR may provide similar performance outcomes as conventional simulation training. This evidence is timely given the rise in the use of virtual learning platforms to facilitate training during the COVID-19 pandemic where face-to-face training may not be feasible.

**Trial Registration:**

The study was registered at ClinicalTrials.gov NCT04330924.

## INTRODUCTION

As key members of interprofessional teams, nurses and doctors play critical roles in assessing and managing patients at risk of clinical deterioration. It is paramount for undergraduate and postgraduate education programs to equip medical and nursing students with the skills needed to respond to patient deterioration.^1^ Facing critically ill and rapidly deteriorating patients can trigger high levels of stress which can either positively or negatively impact clinical performance.^2^ Training of high-acuity events should go beyond acquisition of skills and knowledge to develop one’s ability to cope and withstand stressors.^3^

Educational interventions using high-fidelity simulation have been shown to increase learners’ ability in recognising and managing patient deterioration.^4^ Furthermore, the emotional climate of a stressful clinical event can be replicated using immersive simulation-based learning.^5^ Apart from stress management interventions, LeBlanc (2009) emphasised the need to provide individuals and teams with specific training to optimise performance during stressful events.^6^ The use of interprofessional simulations for team training has gained popularity in healthcare education.^7^ Despite growing evidence of the effectiveness of interprofessional simulations,^8^ logistical constraints such as scheduling challenges and availability of appropriate training venues impede their broad implementation, particularly at the preregistration level.^9^ The use of virtual reality (VR) for team-based simulation training has generated interest among healthcare educators.^10^ A study by Liaw et al. (2020) did not demonstrate any inferiority in team communication skills training using VR when compared to live simulation.^11^ However, given the different training environments between VR and conventional simulation, it is unclear whether VR stimulates similar stress responses as a physical simulation environment.

VR uses computer technology to create an interactive three-dimensional virtual world in which a user or multiple users can experience a simulated environment.^12^ The level of immersion of VR simulations can vary from the use of less immersive desktop VR to the high immersive head-mounted display (HMD) VR. A scoping review has demonstrated the capability of VR in inducing a stress response in participants; repeated exposures to the simulation can reduce stress and improve performance. However, the review was limited to studies which used immersive VR in surgical procedure. There are also limited studies which explore stress responses and performance outcomes in a nonsurgical situation.^13^ Although immersive VR using HMD has gained popularity and become more affordable, desktop VR is still more accessible and scalable to train large groups of learners. Therefore, the aim of this study was to compare desktop VR with face-to-face simulation in stress responses and performance outcomes of a team-based simulation training in managing clinical deterioration. We hypothesised that the physiological stress responses and performance outcomes would be similar between learners in the desktop VR and face-to-face simulation. In addition, the correlations between each stress response and learning outcome were investigated.

## METHODS

### Study Designs, Setting, and Participants

This was a randomised control trial (RCT) using pre-test and post-test study designs. The CONSORT guideline was applied to report the RCT. The study was approved by the University’s institutional review board (NUS-IRB, Ref No: S-17-107). All third- and fourth-year medical and nursing students from the university were recruited through the use of recruitment posters. The sample size was derived based on Cohen’s (1992) sample size table.^14^ To achieve 80% power, a sample size of 64 participants per group was needed to detect a medium effect size for a two-sided independent sample *t*-test with a 5% level of significance.

The participants were recruited and randomly assigned by the research coordinator to either the VR or SP group, and stratified based on healthcare course (medicine or nursing) and year of study (year 3 or 4). Stratified randomisation was utilised to ensure that both groups had comparable characteristics with regard to the type of course and year of study. Allocation concealment was achieved as participants were unaware of their designated group. However, blinding of participants from their allocated group was not possible when they arrived to undertake the simulation for their study intervention. Participants in both groups were put into interprofessional teams, which comprised of two medical students and two nursing students.

### Procedure

Participants were briefed on the study intervention after they provided informed consent. They then watched a short video on interprofessional collaboration and communication, completed both demographic and pre-test questionnaires, and had their baseline blood pressure (BP) taken by the research team. The participants were also given a smartwatch to wear for continuous tracking of their heart rates (HR) before receiving their orientation.

The participants in the SP group were given an orientation to the simulated ward setting, equipment, and a simulated patient in the simulation laboratory. The participants in the VR group were brought into individual rooms with computer desktops to log into the virtual reality platform. The platform was developed by an interdisciplinary team from National University of Singapore using the Unity 5 games engine.^15^ The participants were given an orientation on the virtual environment and instructions on how to communicate with their teammate using headsets. They were also introduced to the patient avatar (controlled by a trained simulated patient) who responded to them in real time using voice and actions (e.g. facial expressions and body positioning), and displayed physiological parameters and clinical features (e.g. lung sound) based on the programmed scenarios.

**Figure 1:**
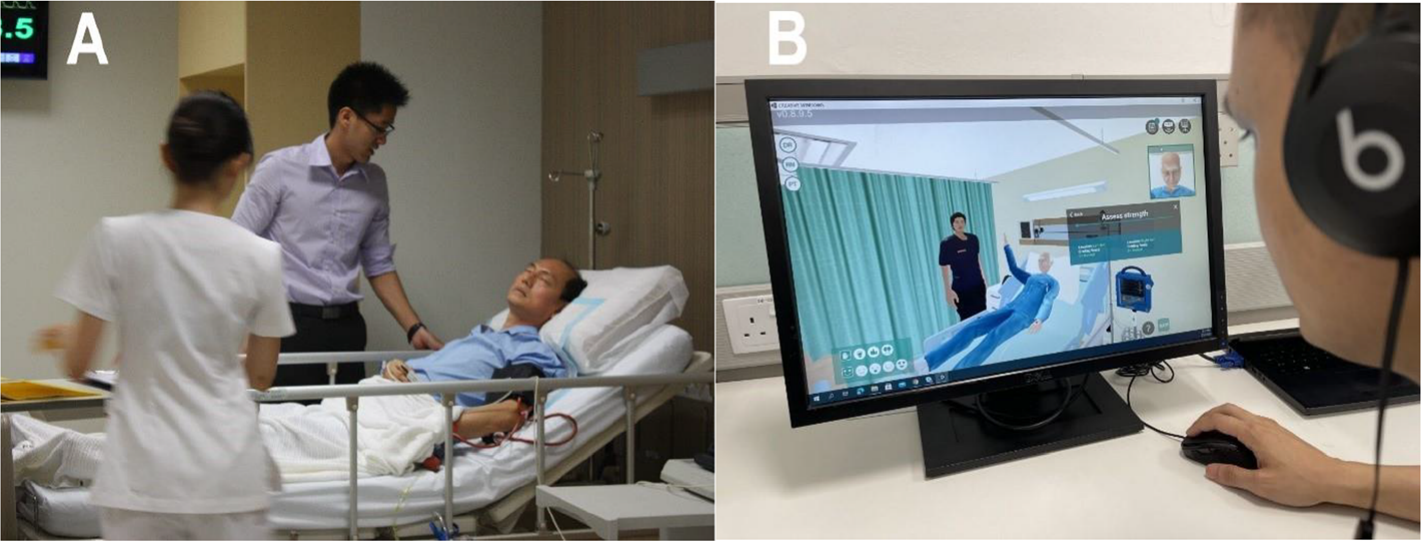
Face-to-face simulation with simulated patient (picture **A**) versus desktop virtual reality (picture **B**). Picture **B** presents the first person’s viewpoint of a nursing student who controls the nurse avatar in a 3D hospital virtual environment. The nurse avatar can communicate with the doctor avatar (controlled by medical student teammate) and patient avatar (controlled by a simulated patient) in real time using headsets. The nurse can perform a health assessment on the patient’s avatar by clicking on the body parts or control panel

The four participants in each group were divided into pairs (one medical student and one nursing student) to take turns to role-play and observe two simulation scenarios. Figure 1 presents the role-play of a scenario by the participants in SP and VR groups. The scenarios included a morning rounds scenario of a patient with early signs of sepsis, and an emergency scenario of a deteriorating patient in septic shock. Each scenario had to be initiated by a nursing student who would perform the initial nursing assessment and management before escalating it to a doctor. Next, the medical student was expected to perform a patient assessment and collaborate with the nursing students to implement treatment plans at the simulated physical and virtual wards. In both groups, the BP of the role-players was taken immediately after the simulation scenario. Each simulation scenario lasted for about 15 to 20 min and was followed by a 30-min debriefing conducted in either a physical or virtual tutorial room by a facilitator.

After completing the post-test questionnaires, the participants undertook a simulation-based assessment to evaluate their clinical performance. They were instructed to work in pairs with their designated partner, provided with a case history to read, and briefed on the simulation setup. The scenario began with the nursing student assessing and managing a patient simulator with signs and symptoms of clinical deterioration. After escalation of any concerns by the nursing participant, the medical student would arrive at the scene to further assess and manage the deteriorating ‘patient’ with the nursing student. The simulation test scenario lasted about 15 min and was video-recorded.

### Outcome Measures

The HR and BP data of each participant were collected to obtain data biophysiological stress responses due to the activation of the sympathetic-adrenal-medullary axis from the release of catecholamine.^16^ HR was continuously measured using a smartwatch. BP was obtained before and immediately after the simulation using a portable electronic BP monitor. All these measures were used in other studies to measure the effect of stress during simulation ^3,17.^

The State-Trait Anxiety Inventory (STAI) and Confidence scale (C-scale) were administered as pre-post questionnaires to measure self-reported stress and confidence levels. The STAI, a 40-item with 4-point scale, has been used as a popular tool in many studies to measure stress during simulation.^18^ The tool consists of two components that measure state and trait anxiety. While state anxiety measures the anxiety at a particular time, trait anxiety measures the general anxiety that respondents feel.^19^ This study reported a Cronbach’ alpha of 0.94–0.95. The C-scale, a 5-item with 10-point scale, developed by Grundy (1993) was adopted to measure participants’ perceived level of confidence in their clinical performance in assessing and managing deteriorating patients.^20^ A high internal consistency of 0.95 to 0.97 was obtained in this study.

The recorded videos of the simulation-based assessment were sent and rated by assessors using a component of the modified Rescuing A Patient In Deteriorating Situations tool. The tool was adapted from another study which was used by the assessors to rate the video-recorded simulation performance of medical and nursing students.^4^ It consisted of a task-specific checklist (2-point scale — not performed or performed) and a global rating scale (9-point scale anchor with descriptors). The application of the tool to the test scenario was validated for content by four medical and nursing academics and clinicians. The inter-rater reliability of this study was tested by two raters who were blinded to the participants’ groupings; ten videos were scored independently. A high intra-class correlation coefficient of 0.91 (95% CI 0.95 to 0.99) was obtained in this study.

### Data Analysis

Descriptive statistics, chi-square tests, and *t*-tests were applied to determine differences in demographic characteristics between the VR and SP groups. Analysis of covariance (ANCOVA) was used to determine differences in biophysiological stress and anxiety scores between groups with baseline scores as covariances. Independent *t*-tests were also used to compare performance scores between groups. In addition, correlational analysis was used to examine the relationship between the study variables.

## RESULTS

One hundred and twenty students, 60 medical students and 60 nursing students, were recruited in the study. Table 1 presents the demographic characteristics of the participants. There were no significant differences in the demographic characteristics of participants between the VR and SP groups. There were also no significant differences in the baseline scores of the study variables including systolic blood pressure (SBP) (*p* = 0.44), HR (*p* = 0.21), psychological stress (*p* = 0.95), and confidence level (*p* = 0.07) between the groups. These results supported the homogeneity of the participants’ demographic characteristics and baseline scores between the groups.

**Table 1:** Demographic Characteristics of Medical and Nursing Students

Courses			VR (*n*=60)	SP (*n*=60)	Overall (*n*=120)
			*n* (%)/*M* (SD)	*n* (%)/*M* (SD)	*n* (%)/*M* (SD)
Medicine	Age		21.53 (1.07)	21.87 (0.97)	21.70 (1.03)
	Gender	Male	15 (50.0)	17 (56.7)	32 (53.5)
		Female	15 (50.0)	13 (43.3)	28 (46.7)
	Year of study	3	14 (46.7)	13 (43.3)	27 (45)
		4	16 (53.3)	17 (56.7)	33 (55)
	Ethnicity	Chinese	27 (90.0)	28 (93.3)	55 (91.7)
		Indian	2 (6.7)	2 (6.7)	4 (6.7)
		Malay	1 (3.3)	0 (0.0)	1 (1.7)
Nursing	Age		22.10 (0.99)	23.20 (3.60)	22.10 (0.99)
	Gender	Male	1 (3.3)	6 (20.0)	7 (11.7)
		Female	29 (96.7)	24 (80.0)	53 (88.3)
	Year of study	3	7 (23.3)	7 (23.3)	14 (23.3)
		4	23 (76.7)	23 (76.7)	46 (76.7)
	Ethnicity	Chinese	26 (87.7)	24 (80.0)	50 (83.4)
		Indian	2 (6.7)	3 (10.0)	5 (8.3)
		Malay	2 (6.7)	3 (10.0)	5 (8.3)

There were no significant differences in the psychological (*F* = 0.43, *p* = 0.51) and biophysiological stress responses for SBP (*F* = 2.73, *p* = 0.10) and HR (*F* = 3.15, *p* = 0.95) between participants in the VR and SP groups after the team training simulation (see Table 2). Within-group comparison showed a significant increase in the SBP from baseline in both VR (*t* = 3.82, *p* < 0.001) and SP (*t* = 6.12; *p* < 0.001) groups. There was also a significant difference in the psychological stress post-test score from the baselines scores in VR (*t* = 5.83, *p* < 0.001) and SP (*t* = 5.58, *p* < 0.001) groups.

**Table 2:** Comparison of Stress Responses Within and Between Groups

		VR group (*n*=60)			SP group (*n*=60)			
		Pre-test	Post-test	Within group	Pre-test	Post-test	Within group	Between group
		*M* (SD)	*M* (SD)	*T*-value (*p* value)	*M* (SD)	*M* (SD)	*T*-value (*p* value)	*F* value (*p* value)
Biophysiological stress								
SBPa	Medicine	116.00 (13.44)	120.87 (14.23)	2.48 (<0.05)	113.00 (11.30)	125.50 (16.85)	4.84 (<0.001)	4.68 (<0.05)
	Nursing	113.50 (13.33)	120.50 (13.81)	2.88 (<0.01)	112.87 (13.42)	120.43 (15.66)	3.86 (<0.001)	0.02 (0.89)
	Overall	114.75 (13.29)	120.68 (13.90)	3.82 (<0.001)	112.93 (12.30)	122.97 (16.33)	6.12 (<0.001)	2.73 (0.10)
HRb	Medicine	83.67 (6.66)	83.78 (6.58)	0.10 (0.92)	84.24 (6.65)	83.88 (6.51)	0.25 (0.81)	0.01 (0.96)
	Nursing	83.92 (7.97)	85.04 (8.39)	0.72 (0.48)	83.84 (7.82)	84.78 (5.82)	0.53 (0.60)	5.99 (0.89)
	Overall	83.79 (7.28)	84.41 (7.51)	0.63 (0.53)	84.04 (7.20)	84.33 (6.13)	0.25 (0.80)	3.15 (0.95)
Psychological stress								
	Medicine	34.43 (7.26)	41.53 (11.71)	3.52 (0.001)	33.57 (8.37)	42.13 (12.04)	4.36 (<0.001)	0.19 (0.67)
	Nursing	38.17 (9.94)	46.6 (11.28)	4.76 (<0.001)	38.8 (11.00)	48.50 (13.30)	3.66 (<0.001)	0.29 (0.59)
	Overall	36.30 (8.83)	44.07 (11.68)	5.83 (<0.001)	36.2 (10.0)	45.3 (13.0)	5.58 (<0.001)	0.43 (0.51)

^a^Systolic blood pressure

^b^Heart rate

As presented in Table 3, between-group comparison using ANCOVA demonstrated no significant difference in the confidence score between the participants in the VR and SP among medical students (*F* = 0.25, *p* = 0.62), nursing students (*F* = 0.10, *p* = 0.76), and overall (*F* = 0.47, *p* = 0.49). Within-group comparison showed a significant increase in the confidence post-test score from the baseline score for the VR group (*t* = 2.79, *p* < 0.01), but not for the SP groups (*t* = 0.60, *p* = 0.55). Similarly, an independent sample *t*-test showed no significant difference in the simulation performance scores between participants in the VR and SP groups for both medical (*t* = 0.75, *p* = 0.45) and nursing students (*t* = 0.08, *p* = 0.94), and overall students (*t* = 0.61, *p* = 0.54).

**Table 3:** Comparison of Performance Outcomes Within and Between Groups

	VR group (*n*=60)			SP group (*n*=60)			
	Pre-test	Post-test	Within group	Pre-test	Post-test	Within group	Between group
	*M* (SD)	*M* (SD)	*T*-value (*p* value)	*M* (SD)	*M* (SD)	*T*-value (*p* value)	*F*/*T* value (*p* value)
Confidence level							
Medicine	28.90 (6.96)	31.83 (9.44)	1.83 (0.08)	30.27 (6.92)	31.87 (9.72)	1.21 (0.24)	0.25 (0.62)
Nursing	28.93 (6.79)	31.77 (6.82)	2.13 (<0.05)	32.37 (8.27)	32.23 (9.68)	0.07 (0.95)	0.10 (0.76)
Overall	28.92 (6.82)	31.80 (8.17)	2.79 (<0.01)	31.32 (7.63)	32.05 (9.62)	0.60 (0.55)	0.47 (0.49)
Simulation performance							
Medicine	-	20.2 (5.36)	-	-	21.3 (5.93)	-	0.75 (0.45)
Nursing	-	22.9 (4.74)	-	-	23.0 (5.16)	-	0.08 (0.94)
Overall	-	21.6 (5.20)	-	-	22.2 (5.58)	-	0.61 (0.54)

The Pearson correlation results demonstrated a significant negative correlation between the psychological stress and confidence level post-test score in the VR (*r* = −0.58, *p* < 0.001) and SP (*r* = −0.35, *p* < 0.01) groups, and overall (*r* = −0.43, *p* < 0.01) groups. No statistically significant correlation was found for the other study variables.

## DISCUSSION

In this study, we found no significant difference in stress responses between participants in the VR and SP groups in terms of recognising and managing simulated clinical deterioration situations. We also found no difference in the level of confidence and clinical performance elicited by the two modalities. Thus, our findings suggest that VR simulation is comparable to conventional face-to-face simulation using SP in terms of inducing stress responses and developing clinical performance to assess and manage clinical deterioration.

A striking finding in our study was the ability of the VR simulation to induce psychological and physiological stress that was almost comparable to the face-to-face simulation using SPs. The use of SPs in immersive simulation which closely resembled a real-life situation was thought to be more stressful and produce higher stress response than VR. According to Finseth et al. (2018), stress can be induced in VR simulation by placing emotional, social, cognitive, and physical demands on users.^21^ In our VR simulation, cognitive demand was created for students to manage a deteriorating virtual patient. The emotional demand was invoked by signs and symptoms of deterioration, including noise (e.g. breathless sound) presented by the virtual patient. The social demand was created by the interactive multi-player virtual environment. While the ability to ensure anonymity using avatars in VR has been found to reduce social anxiety,^22^ the perceived social judgement from avatars may potentially invoke a stress response.^23^ Despite limited physical demands, which involve the movement of wrist and fingers to navigate the virtual environment, the unfamiliarity with the virtual technology could also potentially increase stress levels.^24^ Future studies could examine the effects of an unfamiliar virtual platform on stress levels.

To our knowledge, this is the first study which demonstrates that desktop VR can potentially be as effective as conventional face-to-face simulation for interprofessional team training in the care of a clinically deteriorating simulated patient. A number of studies that examined the effectiveness of VR using HMD demonstrated that the performance outcomes in terms of disaster management were comparable to immersive simulation.^25,26^ Similar effectiveness was reported in our findings with the use of a desktop VR despite its lower physical resemblance to HMD VR. Likewise, studies have found that a virtual patient simulation with low physical fidelity was just as effective as a high-fidelity mannequin-based simulation for training healthcare students’ clinical performance.^27^ Therefore, in congruence with previous studies, our finding showed that immersive simulation may not be essential for developing the desired learning and performance outcomes.^3,27^ Hamstra et al. (2014) recommended for simulation-based education to move away from focusing on the physical resemblance of the simulation to ensure close alignment between the simulation task and the clinical task (i.e. functional fidelity) in order to achieve the learning objectives.^28^

The lack of significant differences in the performance outcomes in our study suggests a close match in task demands between VR and conventional immersive simulation. Despite differences in physical resemblance, we ensured functional task alignment between the two simulation modalities, including the undertaking of orientation, briefing on learning objectives, and experiential learning activities (i.e. role play and debriefing). We emphasised high functional fidelity in the simulation scenarios for both modalities by allowing the medical and nursing participants to interact with each other and with a SP either face-to-face in the immersive simulation or using headsets in the VR simulation. Similar to face-to-face simulation, the participants in the VR scenario were able to assess and manage a patient with clinical deterioration, and received feedback through the patient responses and facilitator-led debrief. As the ability to recognise and manage clinical deterioration involves cognitive tasks, the use of simulation with high functional fidelity was observed to be more important than physical fidelity for developing these cognitive skills.^29^

While this study demonstrated that the anxiety levels were negatively correlated with confidence levels, no association between either physiological or psychological stress response and performance score was found. The influence of stress on performance has been known to be inconsistent as it could lead to poor, better, or no effect on performance. The inverted-U curve, described by Yerkes and Dodson (1908),^30^ was applied to a previous study to support the relationship between stress level and clinical performance.^31^ However, a study on the effect of anxiety on nursing students’ simulation performance by Al-Ghareeb et al. (2019) supported the inverted-U model that was skewed more to the left, indicating that low levels of anxiety may lead to optimal performance and moderate- to high-level anxiety may diminish performance.^31^ Nevertheless, the study highlighted the need for sufficient exposure to simulation training to enable students to cope with stressful situations.^31^

There are limitations in our study. Firstly, the recruitment of volunteers in this study could be a potential source of selection bias as they may exhibit characteristics that differ from the non-volunteers. This could reduce the generalisability of the trial results.^32^ Secondly, although continuous HR measurements via smartphone were measured, the HR data may have been confounded by movement artefacts especially during the immersive simulation. Future studies could consider more reliable HR measurements such as HR variability which determines variation in heart rhythm through QRS intervals.^33^ Thirdly, the use of self-reported measures for psychological stress and confidence level may be subjected to social desirability, and thus caution is needed when interpreting the reported findings. Lastly, although we examined performance outcomes at the individual level, the performance was based on simulation-based assessments conducted immediately after the simulation training. Future studies could investigate the retention of learning as well as the impact of repeated stress on performance over time for the two simulation modalities.

## CONCLUSION

The opportunity for medical and nursing students to experience stress responses during simulation training is crucial in preparing them to cope with stressors involved in the actual care of deteriorating patients. Our study demonstrates that interprofessional team training using desktop VR induced physiological and psychological stress responses among the students, which were similarly observed in the immersive face-to-face simulations using SP. In addition, a well-designed virtual reality environment that focuses on the required functional fidelity to train students in clinical deterioration situations can provide performance outcomes similar to conventional mannequin-based immersive simulations. This evidence is timely given the rise in the use of virtual learning platforms to facilitate training during the COVID-19 pandemic, which has made it challenging for healthcare students to come together and participate in face-to-face simulation training.
